# Polypyrrole/Schiff Base Composite as Electromagnetic Absorbing Material with High and Tunable Absorption Performance

**DOI:** 10.3390/molecules27196160

**Published:** 2022-09-20

**Authors:** Ji Huang, Huiling Gu, Na Li, Hua Yang, Gang Chen, Lizhu Zhang, Chengjun Dong, Hongtao Guan

**Affiliations:** 1 School of Materials and Energy, Yunnan University, Kunming 650091, China; 2 School of Ethnic Medicine, Yunnan Minzu University, Kunming 650500, China

**Keywords:** polypyrrole, Schiff base, microwave absorption

## Abstract

In recent years, Schiff base-related conjugated systems have received extensive attention, but little research has been done in the field of electromagnetic materials. In this work, an organic conjugated system based on polypyrrole/hydrazone Schiff base (PPy/HSB) composites was constructed via a Schiff base synthetic route and their electromagnetic behavior was investigated. The electromagnetic response of PPy/HSB complexes demonstrates fine electromagnetic absorption performance. When the filler loading is 30 wt% in a paraffin matrix, an absorption peak of −43.1 dB was achieved and its effective absorption bandwidth (EAB) was located in the range of 10.88−18.0 GHz. The electromagnetic response behavior of PPy/HSB complexes is explained by models involving electronic structure, multi-polarization and conductive network. The mechanisms of PPy/HSB complexes formation and HSB crystallization are also discussed through the compatibility of PPy/HSB and the structure of HSB. Moreover, the morphology transformation of HSB in the PPy/HSB systems has been studied. This study opens the exploration of organic–dielectric conjugated systems in the field of electromagnetic materials, and significantly broadens the application range of organic–dielectric–dielectric composites.

## 1. Introduction

With the gradual maturity of 5G technology and the popularity of 6G and terahertz (THz) technology in the future, the application of electromagnetic waves has covered all aspects of human life. At the same time, electromagnetic pollution is becoming more and more serious to human beings and nature. Microwave absorbing materials (MAMs) have become a hot research topic in recent years as a barrier between electromagnetic waves and the normal operation of electronic devices [1,2,3]. The electromagnetic wave (EMW) absorption performance of MAMs depends mainly on impedance matching and attenuation capability (magnetic loss and dielectric loss). Impedance matching determines how much electromagnetic waves can enter the material, whereas attenuation capability measures the degree to which electromagnetic waves are lost after entering the material. The absorption capability of a MAM is generally examined by reflection loss (*RL*), named from its measurement method. *RL* is always negative and a *RL* value superior to −10 dB means an effective absorption. The frequency range in which *RL* < −10 dB is called the effective absorption bandwidth (EAB). Recently, wide frequency bandwidth, thin thickness, lightweight nature and strong absorption have been the pursuit direction of efficient microwave absorbers [4,5,6,7].

Conductive polymers (CPs), as one of the typical MAMs, have high sensitivity to modification, controllability of micromorphology and especially flexible modulation of dielectric and conductivity, which gives them a great advantage over metals [8]. Polypyrrole (PPy), one of the most commonly used conductive polymers, has attracted extensive research in the field of electromagnetic absorption [9]. However, according to the free electron theory, conductive PPy has high permittivity, leading to mismatching between the dielectric constant and permeability, to be more precise, a mismatching in impedance, thus affecting its microwave absorption performance [10]. Therefore, many composites composed of PPy and magnetic metal compounds have been investigated, including Fe_3_O_4_ [11], Co_3_O_4_ [12], MoS_2_ [13], ZnFe_2_O_4_ [14], Ni [15], and other designed PPy magnetic metal composites. In these works, the inclusion of magnetic loss makes a significant improvement in electromagnetic absorption performance. The increased magnetic loss mechanism can somewhat broaden the absorption bandwidth. It should be noted that the metal-based magnetic compound also has the disadvantages of high density, low corrosion resistance and large threshold concentration. Compounding or partially compounding PPy with a dielectric medium can compensate for the above disadvantages. Up to now, there have been new advances in PPy composites compounded with other media and good absorption performances have been achieved. For example, Liu et al. obtained graphene/PPy aerogel (GPA) with microstructural changes by compounding PPy nanorods with graphene [16]. Its *RL* can reach a peak value (*RL*_max_) of −51.12 dB at 6.4 GHz, and the EAB reaches 5.88 GHz. Wang et al. obtained rGO/PPy composites by in situ intercalation polymerization of PPy into the interlayer of graphene oxide [17]. Due to the increased polarization ascribed from its special structure, the rGO/PPy composite results in an enhanced response with a *RL*_max_ of −59.2 dB at 3.8 mm and an EAB as wide as 2.3 GHz. Wu et al. [18] prepared three-dimensional polypyrrole and poly(3,4-ethylene dioxythiophene) (PEDOT) composites by a self-assembly method and achieved a wide bandwidth of 6.28 GHz at 2.5 mm with a 5 wt% filling in paraffin matrix. In addition, polyaniline [19], carbon nanotubes [20], SiC nanowires [21] and other dielectric materials were reported to be introduced into PPy to obtain lightweight and high-performance electromagnetic absorbers. Currently, the composite based on organic dielectric materials with PPy provides another strategy to fabricate high-performance EMW absorbing materials. The obtained organic dielectric–dielectric composites possess a light weight and high stability due to their physical similarity [22]. More importantly, a strong microwave absorption capability can be achieved by adopting effective CPs dielectric fillers.

Schiff base compounds are attractive dielectric materials with a wide range of applications in catalysis [23], medicine [24], sensors [25], photochromic [26] as well as corrosion resistance [27]. The molecules of Schiff base compounds contain a −C=N− characteristic group and π-π interactions. The addition of the carbon and nitrogen double bonds endows these Schiff bases with increased polarization loss characteristics. By controlling dopant species and doping conditions, distinctive dielectric loss can be obtained. These features are conducive to optimizing electromagnetic characteristics and regulating impedance matching [25]. Therefore, Schiff base compounds have exhibited promising applications in electromagnetic absorption. Recently, Xu et al. [25] reported a dielectric–dielectric composite by mixing Ag Schiff base complex with three-dimensional reduced graphene aerogel (3D-rGA), which achieved a *RL* of −63.82 dB at a thickness of 2 mm. In addition, Lin et al. [28] prepared polypyrrole nanotube/ferrocene modified graphene oxide composites using an in-situ chemical oxidation method and achieved a *RL*_max_ of −28.73 dB at a thickness of 3 mm. From these works, it is demonstrated that Schiff base compounds could exhibit good EMW absorption performance as dielectric loss materials. However, there are few studies on this class of compounds, especially, the organic dielectric loss and electromagnetic response behavior of Schiff base compounds need to be further investigated. The synergy between Schiff bases and matrixes still needs to be explored. 

In this paper, we report a hydrazone Schiff base (HSB)/PPy composite for electromagnetic absorption. Considering no more mutual repulsion between the pyrrole ring and the N=C double bond, it may be compatible to combine PPy with HSB. Thus, based on the principle of Schiff base reaction, series of PPy/HSB composites with different morphological structures are synthesized. Incorporation of HSB not only increases the contact area with PPy, thereby increasing the interfacial polarization, but also provides a way to adjust the impedance matching due to its lower dielectric constants. Compared with other similar materials, the composite of HSB with PPy gives a good electromagnetic absorption performance and a wider absorption bandwidth. The incorporation process is energy-saving and environmentally friendly. Notably, the present work not only broadens the application of Schiff base analogues, but also explores the electromagnetic response and development of dielectric–dielectric materials.

## 2. Experiments

### 2.1. Chemicals and Materials

Pyrrole monomer (Py), ammonium persulfate (APS), sodium 4-vinylbenzenesulfonate, glyoxal and hydrazine hydrate were purchased from Macklin Biochemical Co., Ltd. (Shanghai, China). All the raw materials were of analytical grade and used without further purification.

### 2.2. Synthesis of PPy

Firstly, 8.82 g Sodium 4-vinylbenzenesulfonate was dissolved in 200 mL pure water and then 2.0 mL pyrrole monomer was dropped in the solution. After the mixture was under stirring in an ice bath for 30 min, 40 mL APS aqueous solution (containing APS 1.80 g) was slowly dropped into the mixture. After the polymerization process was kept for 24 h, the products were centrifuged. Finally, PPy black powder was obtained after the precipitates were dried at 60 °C for 12 h.

### 2.3. Synthesis of HSB

For the synthesis of HSB, 0.6 mL of hydrazine hydrate and 0.6 mL of glyoxal were added dropwise to 60 mL of deionized water at the same time and stirred for 5 h to produce a yellow precipitate. The precipitate was washed by centrifugation with ethanol and deionized water, and dried at 60 °C for 6 h. The as-synthesized product was named as HSB-0.6. In addition, series of HSB products were synthesized and labeled as HSB-0.3, HSB-1.2 and HSB-1.8, respectively, corresponding to the different volumes of hydrazine hydrate and glyoxal.

### 2.4. Preparation of PPy/HSB

**Figure 1:**
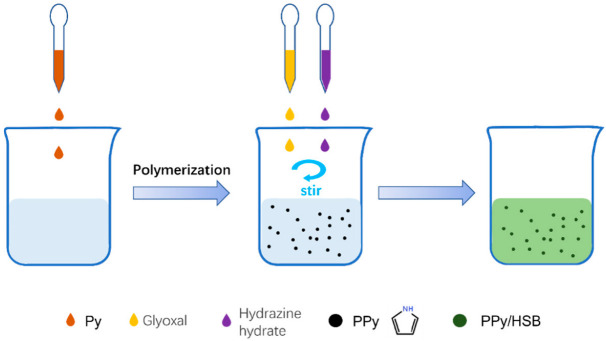
Synthesis route for the PPy/HSB composites.

Firstly, 60 mg PPy powder was mixed with 60 mL water. Then 0.6 mL hydrazine hydrate and the same volume of glyoxal were added at the same time and stirred for 5 h. The color of the solution changed from black to dark green during the stirring process. After centrifugation and drying, the PPy/HSB composite was obtained and named as PPy/HSB-0.6. To verify the effects of HSB contents on the electromagnetic properties of PPy, PPy/HSB composites with different HSB were prepared by changing the volumes of hydrazine hydrate and glyoxal (0.3 mL, 1.2 mL and 1.8 mL) while keeping their volume ratios constant as 1:1. The samples were labeled as PPy/HSB-0.3, PPy/HSB-1.2 and PPy/HSB-1.8, respectively. The synthesis route is shown in Figure 1.

### 2.5. Characterization

The morphology and structures of the composites were determined using field emission scanning electron microscopy (FESEM, Nova NanoSEM 450, Hillsboro, WC, USA). The functional groups on the surface of the samples were studied using Fourier transform infrared spectroscopy (FTIR, Thermofisher Bruck Nicolet iS 10, Waltham, MA, USA) with a scanning range of 4000−400 cm^−1^ (pressed-disk technique). The molecular weight and structure of the samples were determined through High Performance Liquid Chromatography-Mass Spectrometry (HPLC-MS, Agilent 1100 HPLC/TOF, Santa Rosa, CA, USA). The crystal structures of the products were characterized through X-ray diffraction (XRD, Rigaku TTR-III diffractometer, Japan) from 5° to 90° with Cu Kα radiation.

The electromagnetic parameters, including dielectric permittivity (*ε*) and magnetic permeability (*μ*) of the composites, were tested through a vector network analyzer (VNA, Keysight P5004A, Santa Rosa, CA, USA) in the frequency range of 2−18 GHz. The composites were mixed with paraffin wax in the mass ratio of 30 wt% and pressed into a cylindric sample to match the coaxial airline (outer diameter 7.00 mm and inner diameter 3.04 mm). The thickness of the sample is about 2.00 mm. The electromagnetic absorption properties were obtained through transmission line (TML) theory based on the electromagnetic parameters.

## 3. Results and Discussions

### 3.1. Structure Characterization

**Figure 2:**
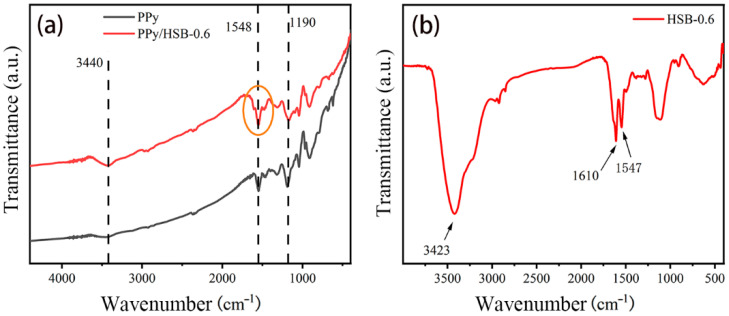
FT-IR spectra of pure PPy, HSB/PPy-0.6 (**a**) and HSB-0.6 (**b**).

The samples were first characterized by FT-IR and HPLC-MS. From the IR spectrum in Figure 2a, the stretching vibration peak of the C=N bond for PPy/HSB-0.6 located at 1548 cm^−1^ appears as a shoulder. From Figure 2b, the C=N stretching vibration of pure phase HSB is located at 1610 cm^−1^ with a shoulder peak locating at 1548 cm^−1^. The vibration peak and the shoulder correspond to PPy and HSB, respectively, which confirms the complexation of PPy and HSB. The N−H stretching bond peak of PPy/HSB-0.6 at 3440 cm^−1^ appears to shift compared to that of pure PPy, which could be due to the existence of the hydrogen bonding (N−H···N). The N−H bonding originally presents only in the imine group on the pyrrole, but the PPy/HSB complex has hydrogen bonding (N−H···N), leading to the relative change of this peak intensity. The two chain length structures of HSB are dominant as seen in the mass spectra in Figure S1, confirming the successful formation of the PPy/HSB complex.

**Figure 3:**
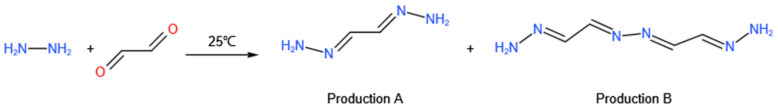
Synthesis route of hydrazone Schiff bases.

Based on the characterization of FT-IR and HPLC-MS, the synthesis route of the hydrazone Schiff bases is shown in Figure 3. Since the chain lengths of Schiff bases are related to the reaction activity, there will be different chain length structures of product A and product B. The mass spectra (Figure S1) show that the content of product A is dominant. The content of longer chain lengths gradually decreases in a certain proportion, due to the result of the competition reaction [29].

### 3.2. Morphology Analysis

**Figure 4:**
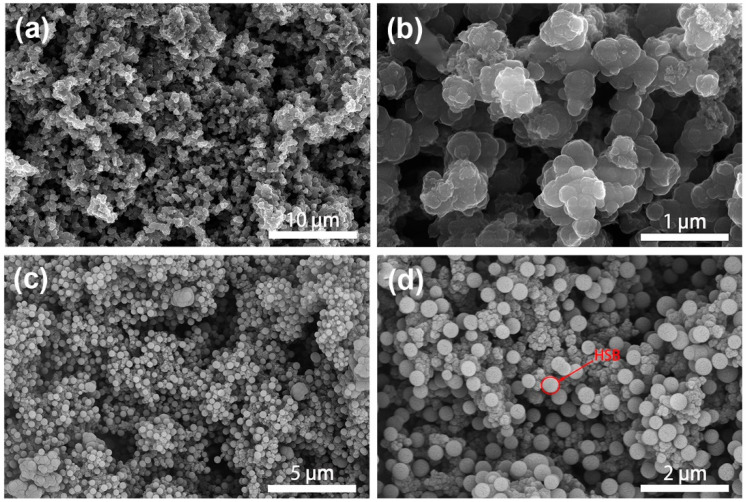
SEM of pure PPy (**a**), HSB-0.6 (**b**) and PPy/HSB-0.6 (**c**,**d**).

**Figure 5:**
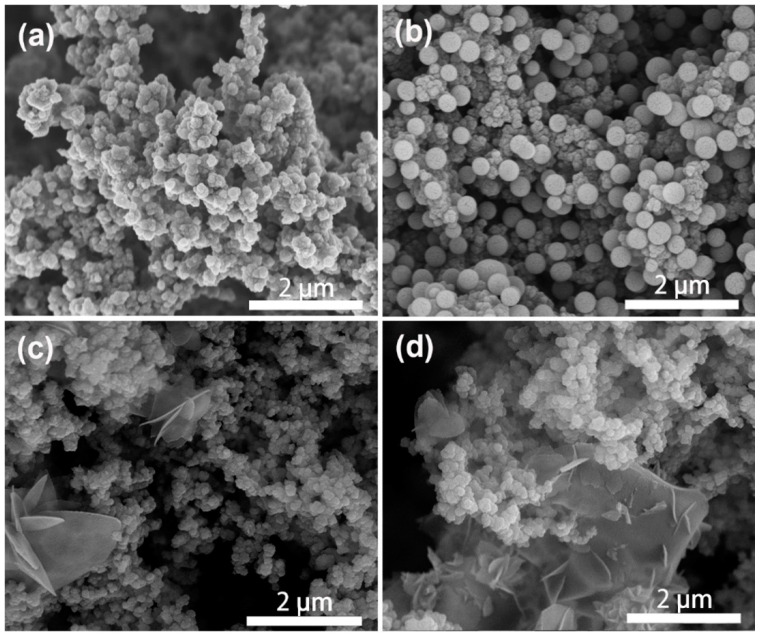
SEM of PPy/HSB composites of PPy/HSB-0.3 (**a**), PPy/HSB-0.6 (**b**), PPy/HSB-1.2 (**c**) and PPy/HSB-1.8 (**d**).

Figure 4 shows the typical morphological structures of pure PPy and PPy/HSB-0.6 composite. As is in Figure 4a, homogeneous PPy prepared by the redox method forms an irregular cauliflower structure with cluster sizes of about 500 nm. The cluster has a smooth surface, which provides conditions for uniform distribution of HSB. Pure HSB displays a morphology of spheres with a size about 500 nm, as shown in Figure S2b. As to the PPy/HSB composites, HSB reacts in situ in the PPy pores as a polar molecule, forming Schiff base spheres by van der Waals forces (orientation, induction, and dispersion forces together), as in Figure 4c–d. Finally, the HSB nanospheres are uniformly dispersed on the PPy surface by hydrogen bonding (N−H···N) [30]. To examine the reactant concentration of HSB on the morphological structures of the final HSB and PPy-HSB composites, both HSB-0.3, HSB-1.2, HSB-1.8 and their PPy-based composites were characterized by SEM, with results shown in Figure S2 and Figure 5, respectively. With the increase of hydrazine hydrate and glyoxal in the HSB synthesis process, the morphology of HSB gradually turns from spheres to flakes.

The growth mechanism of HSB, as well as PPy/HSB composites, can be proposed as in Figure S3. The simple structure and good symmetry of HSB make it easy to arrange in a regular manner, thus forming a dense, stacked structure. However, since its main chain contains carbon and nitrogen double bonds that cannot be rotated, it does not resemble the helical chain conformation of olefins. HSB crystallizes in a chain-axis parallel arrangement, with chemical bonding along with the c-axis and van der Waals forces acting along with the *a* and *b*-axis, which makes the structure anisotropic. HSB forms the basic structural unit of spherical crystals by orderly arrangement of stacked folded chain wafers, and in order to reduce the surface energy, they tend to grow in all directions with certain crystal nuclei as the center, thus developing into spherical aggregates with diameters around 500 nm (Figure S2a). The parallel arrangement causes the HSB polymer to crystallize without cubic crystal system, as evidenced by Figure S4a, from which it is clear that both PPy and PPy/HSB-0.6 show amorphous states.

According to experimental confirmation [31], polymer crystal growth occurs only on the sides of the sheet crystals, i.e., in the two-dimensional direction, and the thickness of the wafer remains constant. The concentration of HSB around the wafer increases as the concentration of the reacting monomer increases or the molar ratio changes, but the wafer growth rate varies due to the different HSB concentrations in the wafer, and the wafer growth is more vigorous in some directions at the end of the reaction. Thus, the morphological structure of HSB gradually changes from spheres to a folded structure, as shown in Figure S2c,d. The XRD result in Figure S4 also shows the increase in the number of characteristic peaks. This is because as the HSB reaction concentration increases, it allows the HSB to stretch in more places where it is easy to grow in an orientation. This corresponds to the results observed in the SEM observation. Compared with spherical structures, the folded flake structures could increase the specific surface area and improve the multiple scattering of the incident electromagnetic waves, but it destroys the conjugate structure of PPy and makes the conductive network ineffective, which would lead to poor absorption performance.

### 3.3. Electromagnetic Properties

The electromagnetic absorption capacity of a MAM is generally evaluated by reflection loss (*RL*), which can be achieved through the TML equation [3,8],

(1) $$RL=20\log\left|\frac{Z_{\mathrm{in}}-Z_{0}}{Z_{\mathrm{in}}+Z_{0}}\right|$$

Here, Z_in_ is the input impedance of the MAM and Z_0_ is the characteristic impedance of free space with a value of 120π Ω. Z_in_ can be obtained from the electromagnetic parameters as follows [3],

(2) $$Z_{\mathrm{in}}=Z_{0}\sqrt{\frac{\mu_{r}}{\varepsilon_{r}}}\tanh\left(j\frac{2\pi f}{c}d\sqrt{\mu_{r}\varepsilon_{r}}\right)$$

where *f* is the frequency, *c* is the speed of electromagnetic wave in free space and *d* is the thickness of the sample. *μ*_r_ and *ε*_r_ are the relative complex magnetic permeability and dielectric permittivity. Generally, *μ*_r_ and *ε*_r_ can be written as *μ*_r_ = *μ*′ −j*μ*″ and *ε*_r_ = *ε*′ −j*ε*″, respectively.

**Figure 6:**
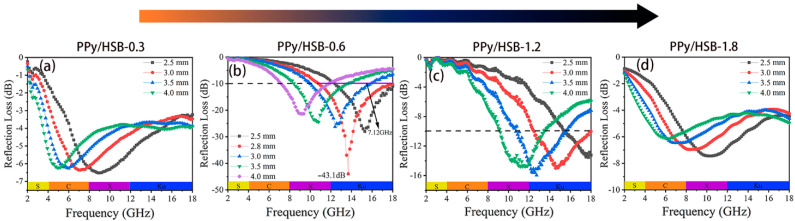
The reflection loss of the PPy/HSB composites. (**a**) PPy/HSB-0.3, (**b**) PPy/HSB-0.6, (**c**) PPy/HSB-1.2 and (**d**) PPy/HSB-1.8.

Figure 6 shows the *RL* curves of PPy/HSB composites with different concentrations of HSB. It is clear that the concentrations of HSB reactants have an important effect on the EMW absorption properties. When the concentration of HSB is greater than 1.2, there is no effective absorption in the whole frequency range. However, the EMW absorption performance of PPy/HSB composites was significantly improved at lower HSB concentrations. In particular, the maximum *RL* value for PPy/HSB-0.6 can reach −43.1 dB at 2.8 mm, and its EAB is as high as 7.12 GHz (10.76−17.88 GHz). With the increase of sample thickness, the absorption peak gradually shifts to lower frequencies, and its effective absorption bandwidth varies in the frequency region of 7.1−17.88 GHz, indicating that the absorption capability of PPy/HSB-0.6 can be modulated by just tuning the matching thickness. Comparatively, PPy and HSB-0.6 only exhibit *RL* values inferior to −10 dB, as in Figure S5. This is due to an impedance mismatching caused by their dielectric being too high or too low, as shown in Figure S5. The obtained PPy/HSB composites possess more superior microwave absorption properties than most previously reported PPy-based absorbers, even at a smaller thickness, as shown in Table 1.

In order to further reveal the absorption mechanism of the PPy/HSB composites, their magnetic loss, dielectric loss and impedance matching are analyzed comprehensively. In general, the dissipation of the incident microwave is composed of dielectric loss and magnetic loss. PPy is a typical dielectric material with negligible magnetic loss. Therefore, dielectric loss is considered as the main attenuation mechanism of PPy/HSB complexes.

**Figure 7:**
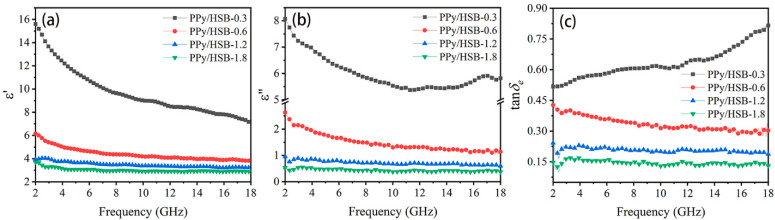
Real permittivity (**a**), imaginary permittivity (**b**) and dielectric loss (**c**) of the PPy/HSB composites.

Figure 7 shows the variation of electromagnetic parameters *vs* frequency for the composites. The *ε*′, *ε*″, as well as the dielectric loss tangents (tan *δ_e_* = *ε*″/*ε*′) of PPy/HSB turn smaller with the increasing frequency throughout the frequency range. It is noteworthy that PPy/HSB-0.6 exhibits the highest *ε*′ and *ε*″ values, indicating its highest dielectric loss properties. As the HSB content increases, the dielectric loss decreases because the introduction of HSB destroys the conductive network of PPy. It thus indicates that the dielectric properties of PPy can be tuned conveniently through changing the contents of HSB components.

As is known, dielectric loss mainly consists of two key factors, i.e., polarization loss and conduction loss [11,37,38,39]. Since PPy is a typical dielectric medium, dielectric polarization is a critical factor that affects the microwave absorption performance. In general, dielectric polarization comes from molecular polarization, atomic polarization, ionic polarization, space charge polarization (with carriers), electron polarization (inner and valence electrons) and dipole polarization (isoelectric positive and negative charge pairs with non-coincident centers) [1,40]. The main polarization modes in GHz frequency are induced polarization and orientation polarization, with orientation polarization playing a dominant role in the attenuation of electromagnetic waves.

**Figure 8:**
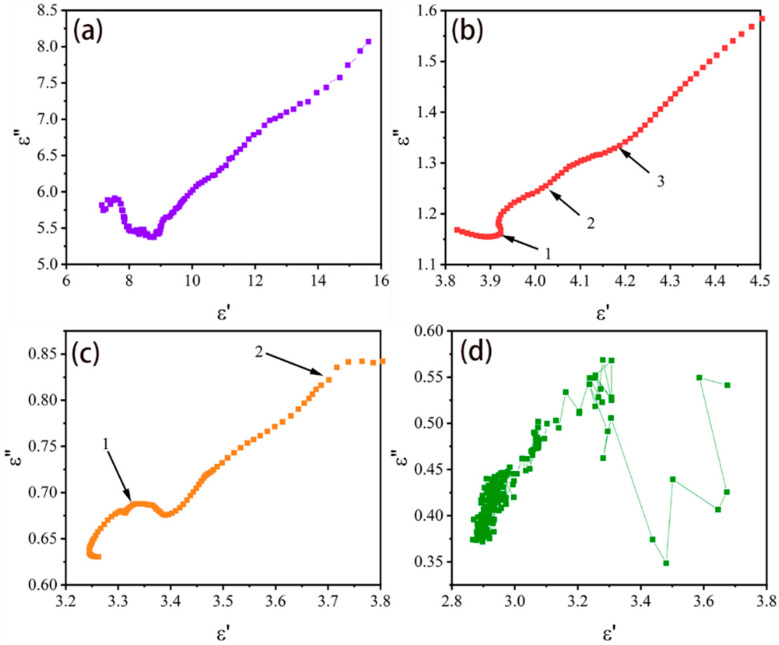
Cole-Cole semicircles of PPy/HSB-0.3 (**a**), PPy/HSB-0.6 (**b**), PPy/HSB-1.2 (**c**) and PPy/HSB-1.8 (**d**).

To further explain the dielectric polarization of the PPy/HSB composites, a Cole-Cole semicircle based on the Debye relaxation is introduced, as shown in Equation (3).

(3) $${\left(\varepsilon^{\prime }-\frac{\varepsilon_{s}+\varepsilon_{\infty }}{2}\right)}^{2}+{\left(\varepsilon^{\prime \prime }\right)}^{2}={\left(\frac{\varepsilon_{s}-\varepsilon_{\infty }}{2}\right)}^{2}$$

where *ε_s_* and *ε*_∞_ are the static dielectric constant and dielectric constant in ultimate frequency, respectively [3]. These semicircles represent the dielectric relaxation processes corresponding to Debye relaxation [41,42,43] and each semicircle represents a polarization behavior [44]. Both PPy/HSB-0.6 and PPy/HSB-1.2 show several Cole-Cole semicircles, as exhibited in Figure 8, indicating there exist complicated polarization mechanisms. The semicircle for PPy/HSB-1.8 and PPy/HSB-2.4 are found to be distorted in Figure 8c,d, indicating that there may be some other processes such as dipole polarization and Maxwell-Wagner relaxation existing in the systems [45].

Doped PPy has carriers, polaritons and bipolaritons [46,47]. When the incident wave comes in contact with PPy, the positive and negative charges of polaritons and dipoles of PPy are separated [48]. The carriers of PPy are excited to generate holes and electrons, leading to dipole polarization and the consumption of electromagnetic waves. For a dielectric material, the different polarity or conductivity of the components on both sides of the interface would cause the charge accumulation at the interface of the two phases under the action of electric field, thus resulting in interfacial polarization. PPy exhibits a cauliflower-like structure, which gives it a high specific surface area and provides conditions for the uniform dispersion of HSB. Therefore, sufficient interfaces can bring about abundant interfacial polarization and thus dissipate more electromagnetic waves.

In addition to dipole polarization and interfacial polarization, conduction loss is also an important factor affecting the absorption characteristics of PPy/HSB composites. The relationship between electrical conductivity (*σ*) and *ε*″ is given as follows [49],

(4) $$\sigma =\varepsilon_{0}\varepsilon^{\prime \prime }2\pi f$$

**Figure 9:**
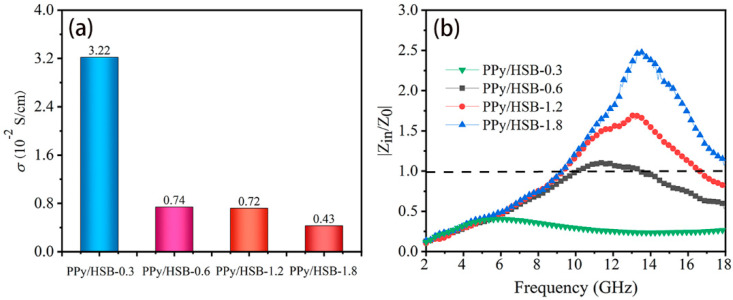
Electrical conductivity (**a**) and impedance matching (**b**) of PPy/HSB composites.

Here, *ε*_0_ (= 8.8542 × 10^−12^ F/m) is the dielectric constant in vacuum and *f* is the frequency. As can be seen in Figure 9a, the conductivity gradually decreases as the HSB concentration increases. Generally, EMW absorbing material with high performance needs to meet two fundamental requirements. The first one is that the electromagnetic waves should enter the interior of the material and not be reflected directly by the surface. In other words, a good impedance matching condition is needed to obtain a good microwave absorption performance. The second requirement is that the MAM must have appropriate attenuation property, i.e., higher conductivity or dielectric loss to transfer the incident microwave energy to other sorts, such as heat energy. Generally, a higher conductivity makes the material more capable of losing electromagnetic waves [50]. However, a much higher conductivity would lead to a strong reflection at the surface of the MAM and thus deteriorate its impedance matching. In this sense, the conductivity must be considered comprehensively with the impedance matching of the MAM.

Figure 9b plots the impedance matching ratios (*z* = |Z_in_/Z_0_|) of the PPy/HSB composites. It is clear that *z* increases with the HSB concentrations, which is in inverse trend with electrical conductivity. As is illustrated in Equations (1) and (2), the balance of permeability and conductivity (increasing the permeability or decreasing the conductivity) can achieve good matching. As the concentration of HSB increases, the permittivity decreases but the permeability keeps constant (Figure S6) without obvious changes, which makes the impedance matching unbalanced.

From Figure 9a, the electrical conductivity of PPy/HSB-0.6 and PPy/HSB-1.2 are nearly the same, but their *RL* values are very different. From Figure 9b, the value of |Z_in_/Z_0_| for PPy/HSB-0.6 is close to 1, whereas the |Z_in_/Z_0_| for PPy/HSB-1.2 is much worse, which explains the phenomenon that PPy/HSB-1.2 has dielectric properties as high as tan *δ_e_* = 0.2, but still exhibits poor EMW absorption performance. The impedance matching conditions of PPy/HSB-0.3 and PPy/HSB-1.8 are even worse, due to the low or high concentration of HSB, which makes the conductive network of PPy broken. The above analysis shows that the excellent microwave absorption performance of PPy/HSB-0.6 comes not only from a good loss mechanism but also from a good impedance matching condition, both of which can be adjusted by introducing HSB into PPy powder.

**Figure 10:**
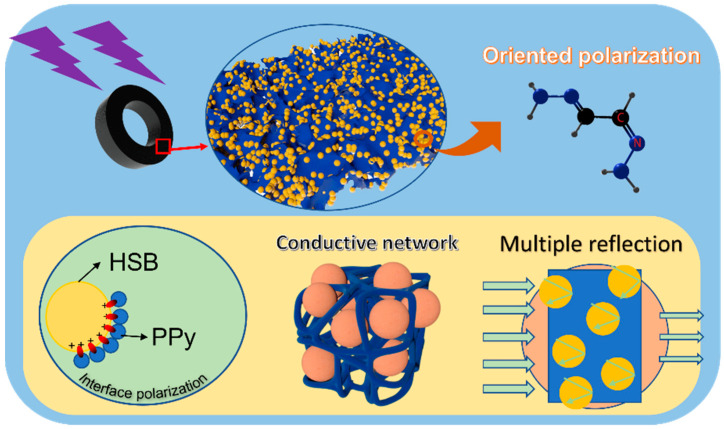
Schematic diagram of the electromagnetic absorption mechanism of PPy/HSB composite.

The EMW absorption mechanism of the PPy/HSB-based composites can be interpreted schematically in Figure 10. Firstly, the composite of conductive PPy and spherical HSB can expand the interface and increase the interfacial polarization. The polarization and related relaxation contribute greatly to the attenuation of the incident wave energy. Secondly, the conjugated structure of PPy makes it easy for electrons to flow and form a conductive network. The resulting induced current can convert the incident electromagnetic wave into other forms of energy for consumption. Finally, the introduction of HSB not only improves the impedance matching of PPy, but also makes it generate multiple dielectric polarization under electric field excitation due to the C=N polar structure therein. The carbon and nitrogen double bond structure in HSB makes itself a permanent dipole moment and results in electromagnetic energy loss when the EMW inters the material [39]. Moreover, the addition of HSB increases the multi-reflection and scattering of electromagnetic waves in the material, which increases the energy consumption and thus leads to the enhancement of absorption. Therefore, the electromagnetic absorption performance of PPy/HSB-0.6 can be improved and the PPy/HSB based composites can be considered as a potential candidate for electromagnetic absorption materials.

## 4. Conclusions

In summary, an organic conjugated system was prepared via a Schiff base synthetic route. Due to the good compatibility of PPy and HSB, PPy/HSB composites were combined successfully through an in-situ polymerization process and characterized for potential application as a dielectric–dielectric electromagnetic wave absorber. With the increase of HSB concentration, the HSB particles transfer gradually from nanospheres to micro-sized flakes with increased crystallinity. The electromagnetic characterization results reveal that the PPy/HSB-0.6 composite exhibits an absorption peak of −43.1 dB with a matching thickness of 2.8 mm. The effective absorption bandwidth reaches as wide as 7.2 GHz. The fine electromagnetic absorption performance can be attributed to the improved impedance matching and increased C=N oriented polarization. The PPy/HSB composite organic conjugated system not only increases the transmission path and enhances the dielectric polarization, but also generates more interfaces to promote the interfacial polarization. This study provides a new idea for the exploration of organic Schiff base systems in the field of electromagnetic functional materials.

## Figures and Tables

**Table 1:** Microwave absorption properties of some typical PPy-based materials reported in paraffin matrix.

Sample	Filling Ratio (wt%)	Thickness (mm)	*RL*_max_ (dB)	EAB (GHz)	Refs
PPy/Co	30	3	−20	7.2	[32]
PPy/graphite	30	2.7	−48	3.4	[33]
PPy-RGO	30	4.0	−49.11	4.88	[34]
PPy-PEDOT	30	2.5	−36	6.28	[35]
PPy@PANI	30	2	−34.8	4.7	[36]
PPy/SMPP	30	3.7	−56.3	6.48	[37]
PPy/SiC nanowires	30	2.5	−16.2	6.52	[38]
PPy/rGO aerogel	30	3	−54.4	6.76	[39]
C@PPy/Ni@Co	30	2	−48.76	5.54	[40]
PPy/HSB-0.6	30	2.8	−43.1	7.12	This work

## Data Availability

The data presented in this study are available on request from the corresponding author.

## Supplementary Materials

The following supporting information can be downloaded at: www.mdpi.com/article/10.3390/molecules27196160/s1, Figure S1: The Mass Spectrometry and Thin Layer Chromatography of HSB; Figure S2: SEM of hydrazone Schiff bases at different reactant concentrations of 0.3 mL (a), 0.6 mL (b), 1.2 mL (c) and 1.8 mL (d); Figure S3: The formation mechanism of HSB; Figure S4: XRD patterns of PPy (a), different HSB (b) and PPy/HSB-0.6 (a); Figure S5: Reflection loss (a, b) and dielectric parameters (c, d) of PPy (a) and HSB-0.6 (b). Figure S6: The real (a) and imaginary (b) permeability of the PPy/HSB composites.
