# Supplementary material for: Polypyrrole/Schiff Base Composite as Electromagnetic Absorbing Material with High and Tunable Absorption Performance
Source: Molecules. 2022 Sep 20;27(19):6160. doi: 10.3390/molecules27196160 (PMC9573551; doi:10.3390/molecules27196160)
Supplement: Supplementary file 1 [file molecules-27-06160-s001.zip › molecules-1917800-supplementary.pdf]

Supplementary Materials

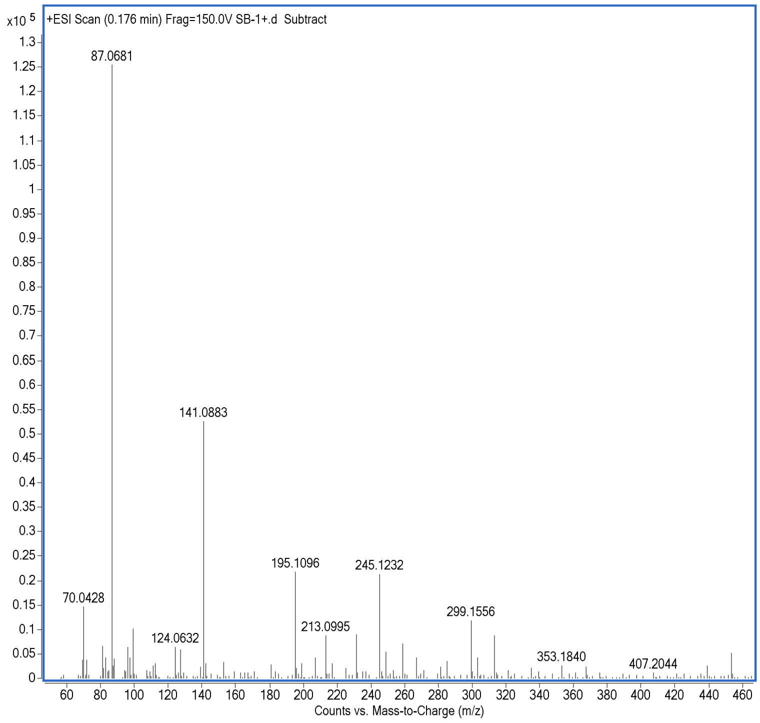

Figure S1. The Mass Spectrometry and Thin Layer Chromatography of HSB

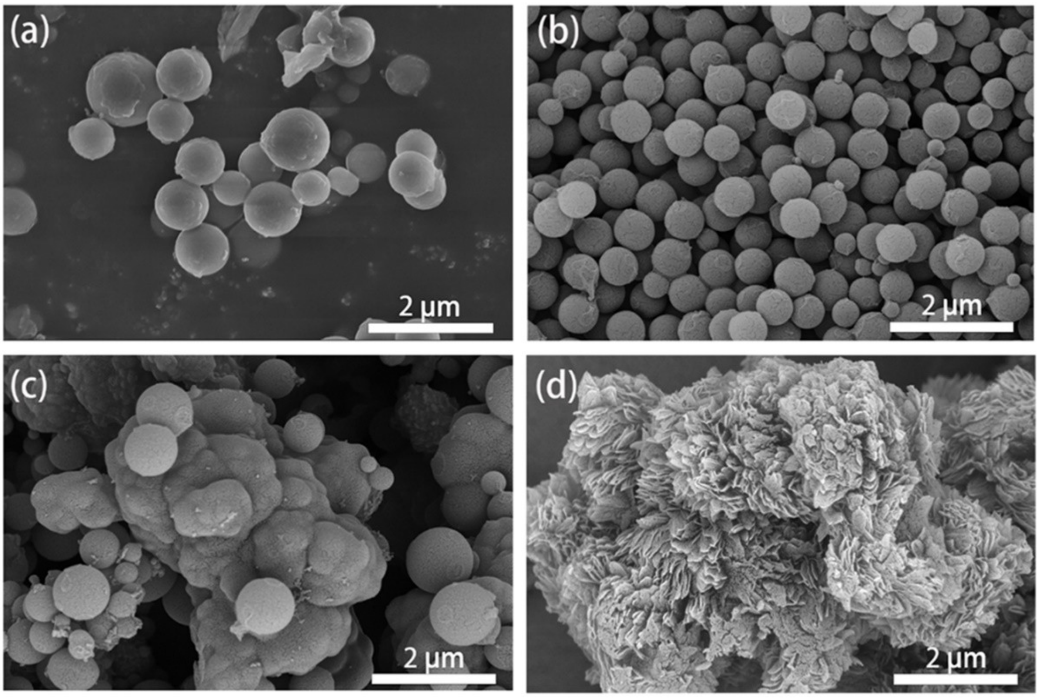

Figure S2. SEM of hydrazone Schiff bases at different reactant concentrations. (a) 0.3 mL;

(b) 0.6 mL; (c) 1.2 mL; (d) 1.8 mL.

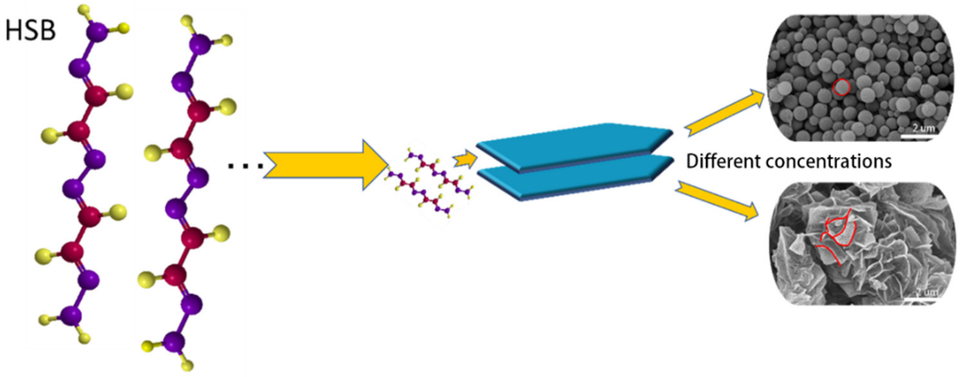

Figure S3. The formation mechanism of HSB.

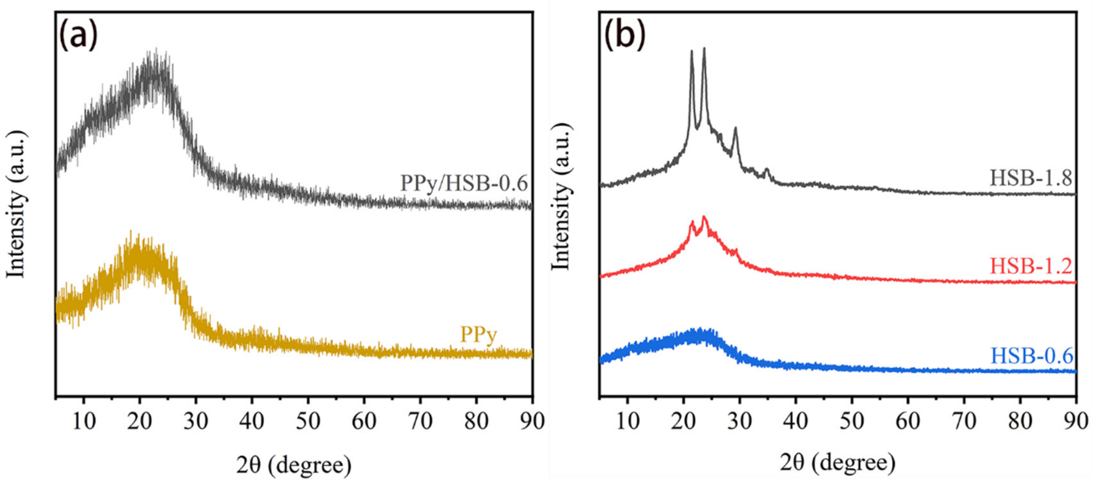

Figure S4. XRD patterns of PPy (a), different HSB (b) and PPy/HSB-0.6 (a).

Unlike the morphology of pure phase HSB, the orientation of HSB in PPy-HSB is not as much as that of HSB. This is because the growth of HSB in PPy is affected by the site resistance, so it does not grow arbitrarily but keeps arranging along some selective orientations.

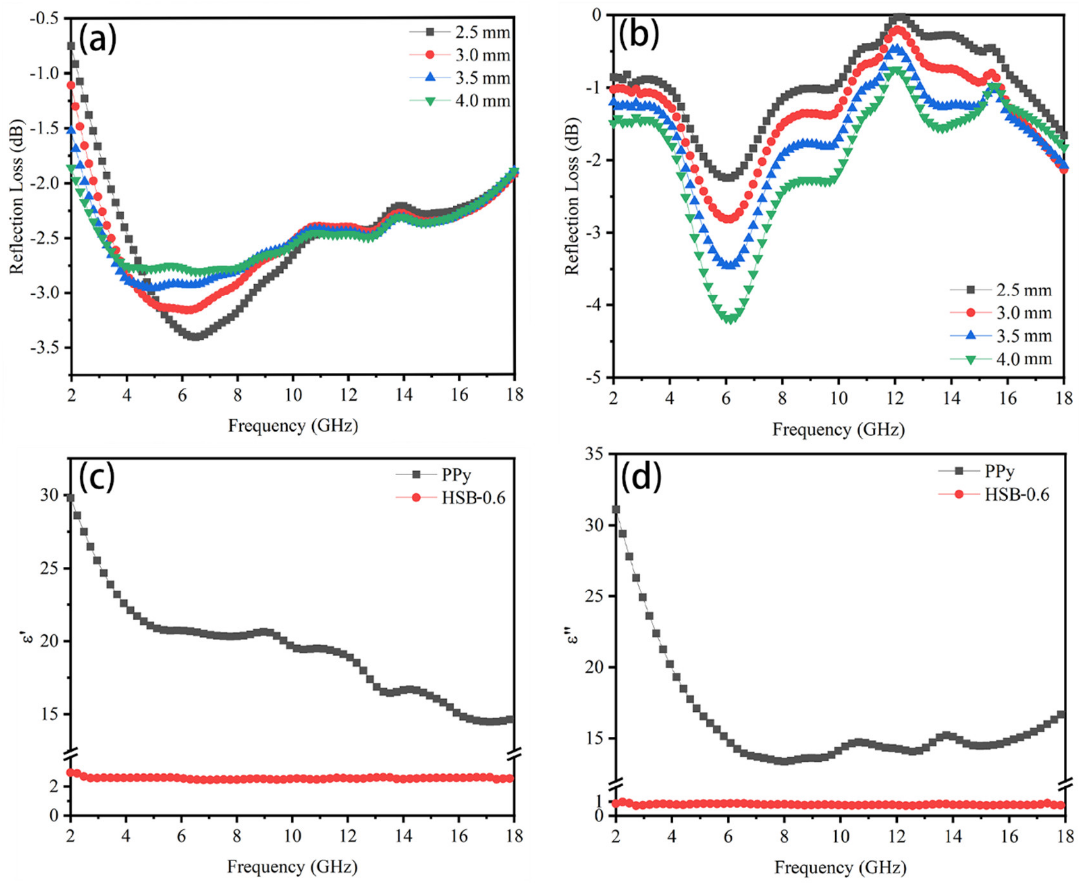

Figure S5. Reflection loss (a, b) and dielectric parameters (c, d) of PPy (a) and HSB-0.6 (b).

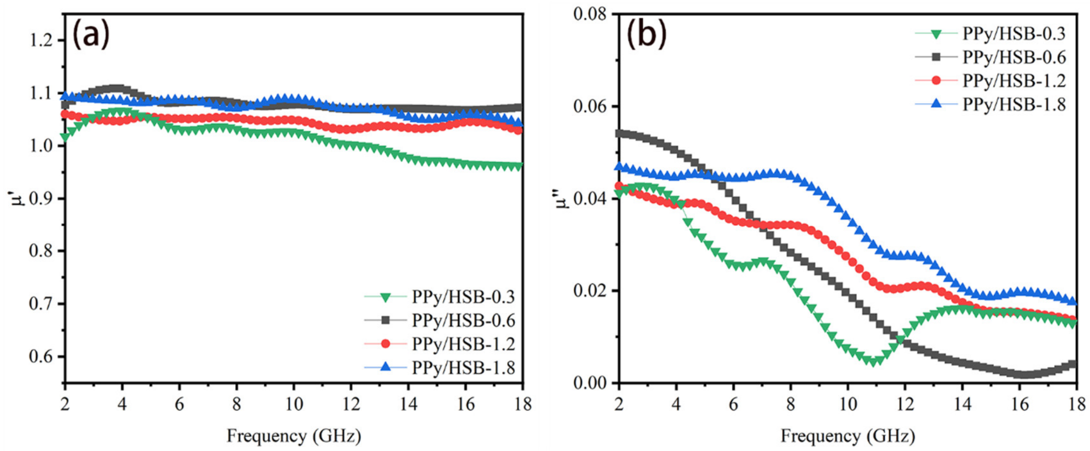

Figure S6. The real (a) and imaginary (b) permeability of the PPy/HSB composites.
